# Sepsis and septic shock after craniotomy: Predicting a significant patient safety and quality outcome measure

**DOI:** 10.1371/journal.pone.0235273

**Published:** 2020-09-17

**Authors:** Jingwen Zhang, Yan Icy Li, Thomas A. Pieters, James Towner, Kevin Z. Li, Mohammed A. Al-Dhahir, Faith Childers, Yan Michael Li

**Affiliations:** 1 Department of Neurosurgery, University of Rochester Medical Center, Rochester, New York, United States of America; 2 Department of Neurosurgery, Huashan Hospital, Fudan University, Shanghai, China; 3 Department of Bioinformatics, University of Nanjing Medical University, Nanjing, China; 4 Department of Neurosurgery, Minimally Invasive Brain and Spine Institute, SUNY Upstate Medical University, Syracuse, NY, United States of America; University of Rome 'La Sapienza', ITALY

## Abstract

**Objectives:**

Sepsis and septic shock are important quality and patient safety metrics. This study examines incidence of Sepsis and/or septic shock (S/SS) after craniotomy for tumor resection, one of the most common neurosurgical operations.

**Methods:**

Multicenter, prospectively collected data from the American College of Surgeons National Surgical Quality Improvement Program (ACS NSQIP) database was used to identify patients undergoing craniotomy for tumor (CPT 61510, 61521, 61520, 61518, 61526, 61545, 61546, 61512, 61519, 61575) from 2012–2015. Univariate and multivariate logistic regression models were used to identify risk factors for S/SS.

**Results:**

There were 18,642 patients that underwent craniotomy for tumor resection. The rate of sepsis was 1.35% with a mortality rate of 11.16% and the rate of septic shock was 0.65% with a 33.06% mortality rate versus an overall mortality rate of 2.46% in the craniotomy for tumor cohort. The 30-day readmission rate was 50.54% with S/SS vs 10.26% in those without S/SS. Multiple factors were identified as statistically significant (*p* <0.05) for S/SS including ascites (OR = 33.0), ventilator dependence (OR = 4.5), SIRS (OR = 2.8), functional status (OR = 2.3), bleeding disorders (OR = 1.7), severe COPD (OR = 1.6), steroid use (OR = 1.6), operative time >310 minutes (OR = 1.5), hypertension requiring medication (OR = 1.5), ASA class ≥ 3 (OR = 1.4), male sex (OR = 1.4), BMI >35 (OR = 1.4) and infratentorial location.

**Conclusions:**

The data indicate that sepsis and septic shock, although uncommon after craniotomy for tumor resection, carry a significant risk of 30-day unplanned reoperation (35.60%) and mortality (18.21%). The most significant risk factors are ventilator dependence, ascites, SIRS and poor functional status. By identifying the risk factors for S/SS, neurosurgeons can potentially improve outcomes. Further investigation should focus on the creation of a predictive score for S/SS with integration into the electronic health record for targeted protocol initiation in this unique neurosurgical patient population.

## 1. Introduction

According to the Third International Consensus Definition for Sepsis and Septic Shock (Sepsis-3) in 2016, sepsis was defined as multiple organ dysfunction caused by infection, and septic shock was defined as a subset of sepsis in which underlying syndromes of complex biochemical, pathological, and physiological alterations are significant enough to add the risk of mortality [1]. Both sepsis and septic shock have a profound impact on patient morbidity and mortality [2].

Sepsis and septic shock has significant psychosocial, cognitive, and physical changes with broad personal and societal impact on patients who are successfully treated [3]. In addition, sepsis and septic shock have a massive financial impact, having recently been reported as the most expensive hospitalized condition in the USA [4]. Sepsis and/or septic shock (S/SS) have also been shown to be increasing in incidence, further expanding their impact as a public health concern [5].

As a result, significant attention has been placed on identifying and managing these syndromes with the Surviving Sepsis Guidelines serving as the national benchmark to improve outcomes [6]. The Centers for Disease Control (CDC) and the Agency for Healthcare Research and Quality (AHRQ), as part of the National Quality Forum, have identified postoperative sepsis as an important contributor to the Patient Safety Adverse Events Composite, or PSI 90 score, which is used to formulate hospital ratings [7].

In the current era of informed consumers, healthcare ratings play an important role as a banner of quality to potential patients. Neurosurgery has embraced this theme of value and quality of care with many publications reporting the socioeconomic and quality aspects of neurosurgical care delivery [8]. Surprisingly, despite all the recent focus on S/SS at the national level neurosurgical literature is lacking on S/SS in the setting of craniotomy for tumor resection. This is a common neurosurgical procedure and an integral part of neurosurgical practice.

Consequently, the primary objective of this study was to present the first detailed assessment of post-operative sepsis and septic shock in patients undergoing craniotomy for tumor resection using a large scale highly powered prospectively collected database. The secondary objective was to analyze the most significant risk factors for S/SS. By doing this study, neurosurgeons can plan ahead to improve outcomes during craniotomy for tumor resection.

## 2. Methods

### 2.1 Inclusion criteria

The American College of Surgeons National Surgical Quality Improvement Program (ACS NSQIP) is a validated prospectively collected, publicly available, peer-controlled database of a random sample of inpatients and outpatients undergoing non-trauma surgery in approximately 400 academic and community hospitals across the United States. This database was used to identify patients undergoing craniotomy for brain tumor from 2012–2015.

Strict standard definitions are used by data extractors to collect data at an institutional level that is submitted by trained personnel. Data collected include preoperative risk factors, comorbidities, procedures performed by Current Procedural Terminology (CPT) code, and postoperative complications occurring within 30 days of the index operation. CPT procedural codes utilized to build our data set include 61510 Supratentorial craniotomy for tumor, 61521 Infratentorial craniotomy for tumor: midline, 61520 Infratentorial craniotomy for tumor: cerebellopontine angle, 61518 Infratentorial craniotomy for tumor: others, 61526 Translabyrinthine approach for cerebellopontine angle tumor, 61545 Craniotomy for craniopharyngioma,61546 Craniotomy for pituitary macroadenoma, 61512 Supratentorial craniotomy for meningioma, 61519 Infratentorial craniotomy for meningioma. 61575 Transoral approach to skull base, brainstem, or upper spinal cord for biopsy, decompression, or excision of lesion.

### 2.2 Outcomes measures

In addition to measuring the occurrence of sepsis and septic shock, other adverse events were also considered to determine their relationship to our primary measures of sepsis and septic shock. Major adverse events were considered to be the infection of the surgical site or organ space, unplanned intubation, pulmonary embolism, occurrences ventilator >48 hours, progressive renal insufficiency, stroke, cardiac arrest, myocardial infarction (MI), and occurrences of deep venous thrombosis (DVT). Minor adverse events were considered to be wound disruption, perioperative blood transfusion, pneumonia and urinary tract infection. We defined an adverse event as the occurrence of at least 1 major or minor adverse event category.

NSQIP tracked mortality, readmissions and reoperation after discharge for the first 30 postoperative days. Return to OR, unplanned reoperation, readmission, and mortality rate were also evaluated as separate outcome measures.

### 2.3 Covariates

The following parameters were analyzed as patient and operative variables: sex, race, age, body mass index (BMI) as defined by the World Health Organization classification scheme, smoking status, American Society of Anesthesiologists physical status classification, functional health status, ventilator dependence, severe COPD, congestive heart failure (CHF), hypertension, disseminated cancer, steroid use, >10% loss bodyweight in last 6 months, pre-operation systemic sepsis, diabetes, dyspnea, bleeding disorders, pre-operation transfusions, open wound infection, wound classification, emergency case and location of tumor whether supratentorial or infratentorial.

### 2.4 Statistical analyses

Statistical analyses were performed using R version 3.4.1. Multivariate logistic regression models were used to determine the effect of patient factors on readmission, return to OR, and adverse events. Sex, smoking and all risk factors significant in the univariate analyses were included in the multivariate model. The statistical difference was established at 0.05.

## 3. Results

### 3.1 Sepsis and septic shock events

A total of 18642 adult craniotomy for intracranial neoplasms were included in the ACS NSQIP between 2012 and 2015. The rate of S/SS in this cohort was 1.97%, specifically with 1.35% sepsis and 0.65% septic shock with the mortality rates listed (Table 1).

**Table 1:** Sepsis and septic shock events.

Event	Brain Tumor	Sepsis	Septic Shock	S/SS
Total No.	18642	251	121	368
Rate (%)	100	1.35	0.65	1.97

### 3.2 Demographic data of study population

The following clinical factors from the ACS-NSQIP data set were analyzed in terms of their relationship to S/SS: sex, race, age, body mass index (BMI), history of smoking, American Society of Anesthesiologists (ASA) physical status score, functional status prior to surgery, ventilator dependent, severe Chronic Obstructive Pulmonary Disease (COPD), congestive heart failure (CHF), hypertension, disseminated cancer, steroid use, >10% loss bodyweight in last 6 months, pre-operation systemic sepsis, diabetes, dyspnea, bleeding disorders, pre-operation transfusions, open wound infection, wound classification and emergency case(Table 2).

**Table 2:** Sepsis and septic shock events and 30-day mortality rates.

Characteristic	S/SS (%)	None (%)	*p* value
Sex			
Female	159(43.21)	9647(52.79)	<0.001
Male	209(56.79)	8627(47.21)	-
Race			
White	262(71.2)	13028(71.29)	0.142
Asian	9(2.45)	534(2.92)	0.596
Black or African American	27(7.34)	1218(6.67)	0.878
Unknown	70(19.01)	3494(19.12)	-
Age			
18–40	50(12.77)	3007(16.21)	0.033
41–60	128(34.78)	7614(41.67)	0.001
61–80	175(47.55)	7058(38.62)	0.007
>81	15(4.08)	595(3.26)	0.586
BMI			
Underweight = <18.5	21(5.71)	1048(5.73)	0.892
Normal weight = 18.5–24.9	96(26.09)	5075(27.77)	0.229
Overweight = 25–34.9	178(48.37)	9524(52.12)	0.019
Obesity > = 35	73(19.84)	2627(14.38)	0.878
Smoke	73(19.84)	3537(19.36)	0.827
NO	295(80.16)	14737(80.64)	-
YES	73(19.84)	3537(19.36)	0.827
ASA classification			
1-No Disturb	49(13.32)	248(1.36)	<0.001
2-Mild Disturb	227(61.68)	4733(25.9)	<0.001
3-Severe Disturb	82(22.28)	10707(58.59)	<0.001
4-Life Threat	3(0.82)	2393(13.1)	<0.001
5-Moribund	7(1.9)	34(0.19)	<0.001
No assigned	0(0)	159(0.86)	-
Functional health status			
Independent	314(85.33)	17472(95.61)	<0.001
Partially Dependent	38(10.33)	634(3.47)	<0.001
Totally Dependent	5(1.36)	89(0.49)	0.063
Unknown	11(2.99)	79(0.43)	-
Ventilator dependent	22(5.98)	190(1.04)	0.000
Severe COPD	36(9.78)	799(4.37)	0.000
Congestive heart failure (CHF)	2(0.54)	57(0.31)	0.475
Hypertension	199(54.08)	6917(37.85)	0.000
Disseminated cancer	93(25.27)	3929(21.5)	0.220
Steroid use for chronic condition	84(22.83)	2715(14.86)	<0.001
>10% loss body weight in last 6 months	13(3.53)	392(2.15)	0.103
Pre-operation Systemic Sepsis			
SIRS	39(10.6)	589(3.22)	<0.001
Sepsis	5(1.36)	28(0.15)	<0.001
Septic Shock	3(0.82)	8(0.04)	<0.001
Diabetes			
Insulin	37(10.05)	764(4.18)	<0.001
Non-Insulin	37(10.05)	1342(7.34)	0.096
Dyspnea			
At rest	3(0.82)	68(0.37)	0.201
Moderate exertion	24(6.52)	657(3.6)	0.007
Bleeding disorders	19(5.16)	354(1.94)	<0.001
Preop Transfusions	6(1.63)	57(0.31)	<0.001
Open wound infection	6(1.63)	149(0.82)	0.113
Wound classification			
1-Clean	355(96.47)	17772(97.25)	<0.001
2-Clean/ Contaminated	2(0.54)	219(1.2)	0.222
3-Contaminated	7(1.9)	221(1.21)	0.285
4-Dirty/Infected	4(1.09)	62(0.34)	0.022
Emergency case	32(8.7)	1166(6.38)	0.130

ASA: American Society of Anesthesiologists, BMI body mass index

### 3.3 Mortality, reoperation and readmission outcomes

Postoperative outcomes are listed in Table 3. The rates of 30-day mortality, patients still in hospital, unplanned reoperation and readmission in patients with S/SS were all significantly higher than patients without S/SS (*P*<0.01). Specifically, the rate of 30-day mortality of patients who had undergone brain tumor resection and developed S/SS was 18.21%, while the rate of 30-day mortality in brain tumor resection patients without S/SS was only 2.14%. The 30-day readmission rate was 50.54% with S/SS vs 10.24% in those without S/SS (Table 3).

**Table 3:** Mortality, reoperation and readmission outcomes.

	S/SS (N%)	None (N%)	*p* value
30-day Mortality	67(18.21)	391(2.14)	<0.01
Still in hospital	47(12.77)	169(0.92)	<0.01
Unplanned Reoperation	131(35.60)	881(4.82)	<0.01
30-day Readmission	186(50.54)	1872(10.24)	<0.01

### 3.4 Incidence of S/SS in different location of the brain

On the basis of CPT code, we were able to assess the frequency of S/SS in supratentorial tumors (except craniopharyngioma) and infratentorial tumors (except craniopharyngioma). The incidence of S/SS in these two groups was 1.84% and 2.66%, respectively (Table 4). As craniopharyngioma has a separate CPT code, it was neither included in the supratentorial tumor group nor the infratentorial tumor group. The incidence of S/SS in craniopharyngioma is as high as 4.04% (4 S/SS out of 99).

**Table 4:** Incidence of S/SS based on location in the brain.

CPT code	Description	Total	S/SS	Percentage	*p* value
61510, 61512	Supratentorial tumor	14572	268	1.84%	0.0019
61518, 61519, 61520, 61575	Infratentorial or posterior fossa	3245	87	2.68%	

### 3.5 Complications associated with S/SS

**Fig 1:**
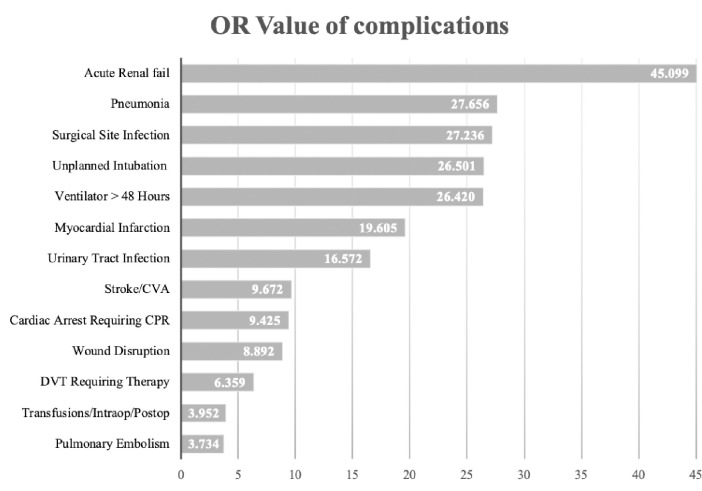
Complications related to S/SS (all *p* values <0.001), the abscissa shows the OR value.

Numerous additional postoperative complications were found associated with S/SS (Table 5). The top five secondary complications associated with S/SS were acute renal failure (OR = 45.1), pneumonia (OR = 27.7), surgical site infection (OR = 27.2), unplanned intubation (OR = 26.5) and ventilator dependence > 48 hours (OR = 26.4). Other complications include wound disruption, urinary tract infection, transfusions, pulmonary embolism, stroke/CVA, cardiac arrest requiring CPR, myocardial infarction as well as DVT requiring therapy (Fig 1).

**Table 5:** Post operative complications associated with S/SS.

	OR	Lower 95% Cl	Upper 95% Cl	*p* value
Minor adverse events				
Wound Disruption	8.9	3.3	19.8	<0.0001
Pneumonia	27.7	21.4	35.5	<0.0001
Urinary Tract Infection	16.6	12.6	21.6	<0.0001
Transfusions/Intraop/Postop	4.0	2.9	5.2	<0.0001
Major adverse events				<0.0001
Surgical Site Infection	27.2	21.0	35.1	<0.0001
Unplanned Intubation	26.5	20.7	33.9	<0.0001
Pulmonary Embolism	3.7	2.2	6.0	<0.0001
Ventilator > 48 Hours	26.4	20.7	33.5	<0.0001
Acute Renal fail	45.1	17.3	117.6	<0.0001
Stroke/CVA	9.7	6.6	13.7	<0.0001
Cardiac Arrest Requiring CPR	9.4	4.8	18.7	<0.0001
Myocardial Infarction	19.6	9.4	41.0	<0.0001
DVT Requiring Therapy	6.4	4.6	8.9	<0.0001

### 3.6 Preoperative/Intraoperative patient factors associated with S/SS after univariate and multivariate analysis

**Fig 2:**
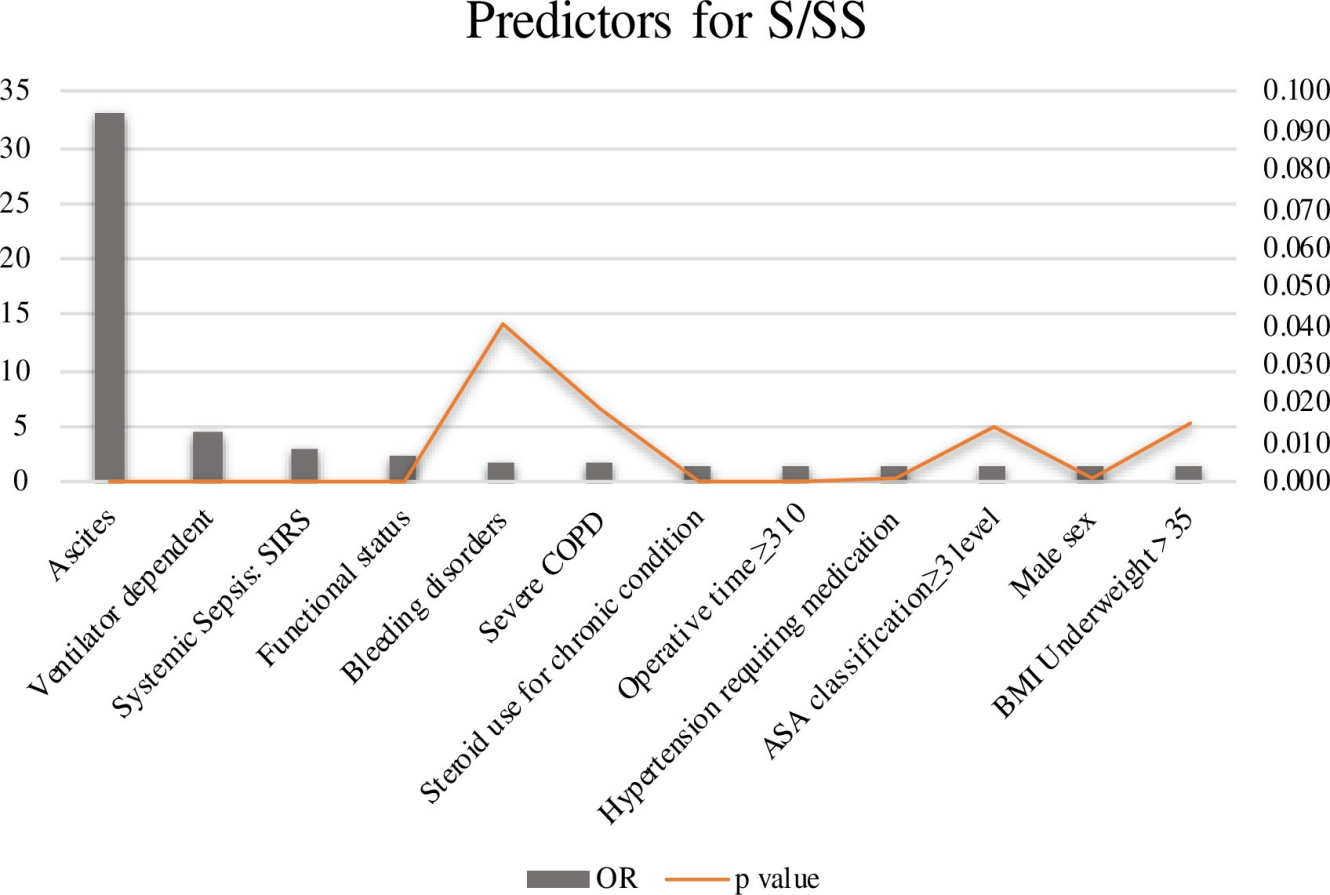
Predictors for S/SS, the abscissa shows the OR value. Y-axis on the left shows OR values, Y-axis on the right shows *p* values.

To discern independent factors of S/SS after craniotomy, a multivariate logistic regression model was used with S/SS as the outcome. Several statistically significant associations persisted (Table 6). Among all the factors, presence of ascites preoperatively was the most significantly associated factor. Additional preoperative factors included ventilator dependence, SIRS, and preoperative functional status. Female sex was associated with an increased odds ratio of S/SS relative to male sex. Bleeding disorders, steroid use, severe COPD, operative time >310 minutes, hypertension requiring medication, ASA class ≥ 3 and BMI >35 were also predictors of S/SS (Fig 2).

**Table 6:** Preoperative/Intraoperative factors associated with S/SS.

Factors	OR	Lower 95% Cl	Upper 95% Cl	*p* value
Male sex	1.4	1.1	1.8	0.001
Ventilator dependent	4.5	2.6	7.3	<0.0001
Severe COPD	1.6	1.1	2.3	0.019
Hypertension requiring medication	1.5	1.2	1.9	0.001
Steroid use for chronic condition	1.6	1.2	2.0	<0.0001
Bleeding disorders	1.7	1.0	2.7	0.041
Ascites	33.0	6.5	136.0	<0.0001
SIRS	2.8	2.0	4.0	<0.0001
ASA classification ≥3 level	1.4	1.1	2.0	0.014
Functional status	2.3	1.6	3.2	<0.0001
BMI Underweight >35	1.4	1.1	1.8	0.015
Operative time ≥ 310	1.5	1.2	1.9	<0.0001

## 4. Discussion

Postoperative incidence of S/SS in patients undergoing craniotomy for brain tumor was 1.97% and these patients tended to have poor outcomes. Additionally, patients with infratentorial tumors were more likely to suffer from S/SS than those with supratentorial tumors(*P* = 0.0019). Finally, we found many complications that were closely associated with S/SS and endeavored to identify common predictors of S/SS.

Sepsis and septic shock are increasing in incidence, highlighting their impact as a public health concern [2]. Complex disorders resulting from dysregulated host responses to infections, they are correlated with acute organ dysfunction [3]. Our results show that the mortality of patients who develop postoperative S/SS within 30 days of operation was 18.21%. In a series of large-scale studies on sepsis of any etiology, the mortality of sepsis was 25–30%, with in-hospital mortality reported as 40–60% [9–12]. As such, preoperative understanding of which patients constitute high-risk for post-operative S/SS will alert the clinician such that every effort can be made to prevent S/SS and its related complications.

Although the specific tumor location is not available in the ACS NSQIP dataset, we were able to separately assess supratentorial tumors, cerebellopontine angle (CPA) tumors, brainstem tumors, and other infratentorial tumors according to CPT code. Of 811 CPA tumors, only 20 cases of S/SS occurred, and of 16 brainstem tumors there was only 1 case of S/SS. Given such low numbers, we were unable to analyze these separately and, as such, we stratified these into the infratentorial tumor group. A recent study by Uzuka T et. al [13] reported no significant difference in the frequency of surgical site infection (SSI) between supratentorial and infratentorial malignant brain tumor. This study, however, may have been underpowered as we found a significantly higher incidence of S/SS in our infratentorial tumor group when compared to the supratentorial tumor group (*p* = 0.0019). Finally, we found the incidence of S/SS in craniopharyngioma to be as high as 4.04% (4 S/SS out of 99), but given the low sample size, we were unable to draw more stringent conclusions.

Our study found that pneumonia, urinary tract infection (UTI) and SSI were highly associated with S/SS. This is consistent with previous literature on spinal tumors, hip fracture and joint arthroplasties [14–16]. These postoperative factors could indicate a potential source of infection. Consequently, patients who are diagnosed with these complications post operatively should be closely monitored for development of S/SS which can lead to more efficient and effective treatment.

The most associated postoperative complication associated with S/SS in this study was acute renal failure. That being said, the connection between S/SS and acute renal failure has been described in the past and is a known tenet of the treatment of septic shock [3]. Furthermore, this study also highlights many other complications (Table 5) that are associated with S/SS. Although the directionality of this association cannot be fully determined, detection of these complications can alert the clinician to optimize the patient against the development of S/SS and its complications.

A preoperative diagnosis of ascites was the factor most associated with the development of postoperative S/SS in this study, with OR of 33.0, far more than any other predictors. Other papers have also discussed ascites, however, found ORs no more than 5 [14, 17]. Our data would suggest that serious consideration should be had regarding treatment of ascites prior to undertaking craniotomy for tumor resection.

Preoperative mechanical ventilation, SIRS, functional status, bleeding disorders, as well as severe COPD were all significant preoperative factors associated with S/SS for craniotomy for brain tumor resection. These results are similar to previous studies [14, 17, 18]. Additionally, steroid use for a chronic condition has been shown to be closely related to the development of both postoperative infections [19–21] and postoperative S/SS [17], which is consistent with our data. This is likely due to the potential to induce immune-suppression and adrenocortical suppression of corticosteroids [22]. Clinically, these predictors can be useful for neurosurgeons and neurocritical care specialists to provide preventative S/SS care with an evidence-based approach.

Other predictors, such as operative time greater than 310 minutes, male gender, hypertension, BMI as well as ASA classification, were also found to be associated with the S/SS in this study. Although many of these have been reported in a number of papers [10, 17, 23–27], their cohorts did not focus specifically on craniotomy on brain tumor resection. Identification of these factors warrants consideration of strategies to improve efficiency and decrease the amount of time a patient is on the operating table and may lead a clinician to consider medical optimization of hypertension, BMI and ASA classification prior to elective operations if possible.

Multiple literature reported that patient age, diabetes, and smoking status are predictors for the development of postoperative S/SS [17, 23, 28]. Specifically, Chen C et al. showed that patients with diabetes undergoing craniotomy for tumor had an increased incidence of meningitis than those without [29]. However, the majority of the remaining publications demonstrating associations between these factors and S/SS were not in patients undergoing surgery [30]. In this study, we found a statistically significant association between these three factors and S/SS in a univariate analysis. However, when a multivariate model was applied, their p values dropped from the level of predetermined significance. Generally, surgeon preference is for patients to quit smoking and obtain strict blood glucose control whenever possible prior to operative intervention for a multitude of benefits. Though or data does not show a link between these variables and the development of S/SS, given the widely accepted benefits of glucose control and smoking restriction, this data is unlikely to result in change of practice for clinicians in general.

Given our analysis of the ACS NSQIP data, the following recommendations can be made regarding prevention and control of S/SS in patients undergoing craniotomy for tumor resection. The first is, whenever possible, to control the preoperative factors listed in Table 5. Additionally, all efforts to increase efficiency intra-operatively should be made in high-risk patients to avoid the complications that arise from extended surgical time. Finally, patients that are diagnosed with the post-operative complications such as UTI, SSI, pneumonia and the others listed above should be monitored closely for the development of S/SS such that early detection and treatment could occur.

There are also some limitations to this study. First of all, the ACS NSQIP dataset involves outcomes only up to 30 days and as such, only allows analysis of the short-term morbidity and mortality in these patients. Additionally, the ACCP/SCCM Consensus Conference Committee definitions of sepsis, severe sepsis, and septic shock [31] are classified into sepsis and septic shock in the NSQIP definitions. Consistent with our experience that it is difficult to distinguish severe sepsis from septic shock, NSQIP data set classified patients with severe sepsis into the septic-shock category. Moreover, the application of antibiotic regimens on patients is not available in the NSQIP data set, so we cannot qualify insight such highly specific topics. Finally, the specific neurosurgical options, such as tumor size, as well as surgical approach is not available in the ACS NSQIP dataset and as such, our analysis is strictly focused on the still fairly specific subgroup of patients undergoing craniotomy for tumor resection.

Nevertheless, the large number and multicenter nature of the NSQIP provided data with an enormous sample size for statistical analyses and decrease potential bias. Moreover, this study provides clinicians information to better predict which patients may be at a higher risk for S/SS after craniotomy for intracranial tumors.

## 5. Conclusion

This study reports that there is a significantly high rate of mortality, return to the OR within 30, days and 30-day readmission in patients with S/SS. Several risk factors for S/SS after craniotomy for brain tumor resection were also identified. These results may be useful for surgeons in clinical settings to develop risk stratification and therefore make evidence-based medicine decisions to minimize the preoperative, intraoperative and postoperative factors that are associated with S/SS including medicine use, the timing of surgery, or preoperative antibiotic prophylaxis.

## Supporting information

S1 Data (CSV)

S1 File (PDF)

S2 File (PDF)

S3 File (PDF)
