# Supplementary material for: Sepsis and septic shock after craniotomy: Predicting a significant patient safety and quality outcome measure
Source: PLoS One. 2020 Sep 17;15(9):e0235273. doi: 10.1371/journal.pone.0235273 (PMC7498000; doi:10.1371/journal.pone.0235273)
Supplement: S1 File — (PDF) [file pone.0235273.s002.pdf]

UNIVERSITY of ROCHESTER

APPROVAL OF MODIFICATION

March 25, 2019

The RSRB approved the following submission on 3/25/2019. This approval is effective as of 3/25/2019.

This study is approved until to 11/26/2019 inclusive.

Type of Review:	Modification
Title:	Clinical outcome and Complication Prediction of Brain and Spine Surgery Using Machine Learning Model
Investigator:	Yan Li
Submission ID:	MOD00001710
Funding:	None
Groups Notified:	Subjects do not need to be notified, or no local subject enrollment
Notification Methods:	None
Documents Approved:	Protocol_v8.pdf

As the Principal Investigator, you are responsible for ensuring compliance with Policy 901 Investigator Responsibilities. Also, any unanticipated problems involving risks to subjects or others (including unexpected deaths, hospitalizations or serious injuries, breach of confidentiality, loss of privacy) must be reported according to Policy 801 Reporting Research Events.

Association for the Accreditation of Human Research Protection Programs, Inc. Full AAHRPP Accreditation
