# Supplementary material for: Sepsis and septic shock after craniotomy: Predicting a significant patient safety and quality outcome measure
Source: PLoS One. 2020 Sep 17;15(9):e0235273. doi: 10.1371/journal.pone.0235273 (PMC7498000; doi:10.1371/journal.pone.0235273)
Supplement: S2 File — (PDF) [file pone.0235273.s003.pdf]

Clinical outcome and Complication Prediction of Brain and Spine Surgery Using Machine Learning Model

Yan Michael Li, MD, PhD, Jiebo Luo PhD, Dongmei Li PhD, Xiang Liu MD, PhD and Y Icy Li, PhD
Department of Neurosurgery and Oncology
University of Rochester Medical Center
601 Elmwood Ave., Box 670, Rochester, NY 14626

1. PURPOSE OF THE STUDY AND BACKGROUND

1.1. Purpose of the study

We want to develop an online machine learning model system that calculates an individual's risk of developing complications from spine and brain surgery. This model will be constructed based on NSQIP national database and then be further validated using de-identified clinical data from University of Rochester Medical Center (URMC). This online system will help patients and doctors providing a systematic, evidence-based method for estimating risks of post-operative complications faced by individual patients that undergo surgery. It will provide better understanding of the continuum of brain and spine care, perform early individualized intervention to reduce the readmission and reoperation and reduce the cost for complication and reoperation.

1.2. Background

The utilization of surgical therapy of disorders of the spine and brain has been increasing dramatically in the past decades (Matz et al., 2016). New York State on average conducts about 200 spinal and brain surgeries per 100,000 people per year. Despite this trend, a paucity of information examining the issues surrounding the safety of these procedures remains. Existing data are limited by inclusion of small sample sizes, single institution experiences, and selected patient populations (Machado et al., 2016; Moisi, Page, Gahramanov, & Oskouian, 2015). Further, few studies exist comparing the various surgical approaches and the outcomes associated with them (Piper et al., 2017).

This data is important to identify patients at risk, allocate healthcare resources, and adequately inform health care providers and patients of such risks and direct future research to improve perioperative outcomes. Additionally, Spinal instrumentation and fusion is one of the important surgical options for spine diseases, so a great many patients with spine surgery may require spine fusion. Based on the 220% increase in spinal\brain surgery over the last decade, increased rates of reoperations due to surgeries is hypothesized. Based on the literature, it is hypothesized, that patient age, payor, surgeon, length of stay and the region where spine and brain care is acquired greatly influences the patient's chances for clinical outcome and complication(Emblem et al., 2015; Obermeyer & Emanuel, 2016; Senders et al., 2018).

Making difficult decisions about whether to have a potentially risky surgery will get easier for doctors and patients. Our online system will allow anyone with a computer and an internet connection to punch specific information into a web page to find out potential outcomes before ever going under the knife.

2. STUDY DESIGN

2.1. Overview

To assess the complications and outcomes of patients undergoing spine and brain surgery, from simple decompression to spine revision and complex craniotomy, we will extract pre-operative

factors from the National Surgical Quality Improvement Program (ACS NSQIP) national database and use advanced machine learning algorithms to construct a predictive model, which will identify the risk of complications and cost for complication, readmission, reoperation mortality and long term outcomes. The results will be further validated using de-identified medical record. Many of these outcomes and complication can be predicted before surgery.

2.2. Rationale for Study Design

New York State on average conducts about 200 spinal and brain surgeries per 100,000 people per year. It is important to identify patients at risk, allocate healthcare resources, adequately inform health care providers and patients of such risks and direct future research to improve perioperative outcomes.

3. CHARACTERISTICS OF THE RESEARCH POPULATION

3.1. Subject Characteristics

This study will be a longitudinal retrospective study. The patients undergoing spine and brain surgery (i.e. cervical, thoracic, and lumbar surgeries) from the National Surgical Quality Improvement Program (ACS NSQIP) database between the years of 2000-2015 will be identified for constructing predicted model. NSQIP database is publicly available. We have got the de-identified data set from NSQIP database. Then the results will be further validated using clinical data from URMC dataset during 2000 to 2017.

NSQIP database compiles data from randomly selected surgical patients at more than 517 institutions in the US, will be used for this study. This database includes more than 150 prospectively collected perioperative variables about included patients. Trained nurses abstract these data from medical records, operative reports, and patient interviews. Postoperative data are collected for 30 days after surgery for all patients, including discharged patients (Khuri et al., 1998). We have got the de-identified NSQIP dataset from 2000 to 2015. There are about 300,000 spine and brain records and the various include patient preoperative, postoperative factors and outcomes.

We will use a de-identified dataset of the spine and brain patients from URMC. We will apply the de-identified dataset from department of Clinical & Translational Research. The de-identified dataset will not include the patients' names, medical record numbers, or dates (except for the year) and will include only preoperative, postoperative factors and outcomes. There are about 50,000 spine and brain records.

3.2. Inclusion and Exclusion Criteria

Inclusion Criteria: Our research population will be derived from the patients included in these databases that are classified under Current Procedural Terminology (CPT). The patients used these codes will be decided into our predict model. The criteria are designed for patients underwent spine and brain surgery. The following Current Procedural Terminology (CPT) codes were utilized to place a patient: from 61000 to 64999 and 22010 to 22899.

4. METHODS AND STUDY PROCEDURES

We will extract patient and surgical factors associated with outcomes, stratified by primary

diagnosis and primary procedure from multi-modality advanced national databases (Memarian, Kim, Dewar, Engel, & Staba, 2015). We will use univariate chi-square analysis and multivariate logistic regression models to fuse the deep anisotropic features into a dense and isotropic form using SAS and R. They are meaningful features in the prediction model. We will also determine the total number of patients that have had more procedure. Then, we will use machine learning algorithms such as the margin-based classifiers or decision tree to contract robust, unique predictive model.

4.1. **Efficacy Assessments**

The novel model will be further validated using de-identified clinical data from URMC database during 2000 to 2017. We considered a quantitative synthesis to be inappropriate due to the heterogeneity in neurosurgical applications. A qualitative synthesis of results and assessment of risk of bias on outcome, study, and review level is provided by means of a narrative approach. However, to summarize the findings in some quantitative form, the median accuracy and area under the receiver operating curve (AUC) of the prediction performance were calculated for all studies, and the median absolute improvement in performance was calculated for all studies comparing machine learning models versus traditional logistic regression. Accuracy refers to the proportion of correct predictions among the total number of predictions, and the AUC corresponds to the probability that a binary classifier will rank a randomly chosen positive instance higher than a randomly chosen negative one.

5. RISK/BENEFIT ASSESSMENT

5.1. **Potential Risks**

There is minimal to no potential risk for privacy and confidentiality of patient records to be violated. We are requesting de-identifiable dataset from NSQIP database and de-identifiable clinical dataset from URMC database. We would like to predict the potential outcomes before ever going under the knife and diagnosis to which variables influence the rate of reoperations and mortality. We will not get any identified information. This study will protect and secure patient health information and will not influence the care of these patients in anyway.

5.2. **Protection Against Risks**

Multiple levels of protection with both the personnel involved and within the computer software have been established to protected potential breaches of confidentiality. Only Dr. Yan. Michael Li, and Dr. Y Icy Li will be involved in analyzing the de-identified information. Also as researchers, we are blinded to the vulnerable populations and thus cannot bias our studies. The patients will not be directly contacted. Only statistical data such as p-values, odds ratios, and frequencies will be released. All data presented, will be presented in a HIPPA identified fashion, where no patients can be easily identified. Levels of security are accomplished with password protections of the database filed, saved on an encrypted server.

6. CONFIDENTIALIATY OF DATA AND INFORMATION STORAGE

The de-identified data will be maintained without an expiration date. All study information will remain locked in either PI's office or the Department of Neurosurgery. Only the study personnel will have access to the data. Patient confidentiality will be maintained throughout the study. None of the information collected will be revealed to anyone outside the study. When results of this study are reported the patients will not be identifiable. The database of de-identified information will be stored on the University of Rochester Center's servers. The server is protected by a CheckPoint Firewall that limits the source and type of traffic coming into the institution. The University imposes restrictions on network protocols to reduce the

risk of various common vulnerabilities. Other security measures including data only saved on an encrypted network server, modifying the screen saver timeout to 10 minutes.

7. DATA ANALYSIS AND MONITORING

7.1. Sample Size Determination

We will identify patients based on Current Procedural Terminology (CPT) and International Classification of Diseases-Ninth Revision-Clinical Modification (ICD-9 and 10) codes to analyze the spine and brain surgery (i.e. cervical, thoracic, lumbar surgeries and craniomoty) When data is presented, it will be de-identified and patients will not be able to be identified directly or indirectly for the data presented. We would like to look at factors such as age, gender, and diagnosis to determine whether or not those variables influence the outcome and

7.2. Planned Statistical Analysis

We will establish a predictive model through advanced machine learning algorithms to identify potential risk of developing complications based on the associated significant pre-operative factors of patients undergoing spinal surgery. A medical application of a model could be outcome prediction in patients with spine surgery. Supervised learning algorithms are trained on historical patients for whom the length of survival is known. The model is too complex, because containing too many features relative to the number of cases, the model could overfit the data characterized by predicting noise. Overfitting can mean including significant features that are identified using univariate and multivariate logistic regression models. This reduces the prediction error in the training set but at the cost of a reduced generalizability to previously unseen data.

To overcome this problem, a model should include a validation procedure. A basic technique is to use part of the data for training and judging the model's performance on the remainder, or test set. There are 2 competing concerns with this approach. Using a very high proportion of data for training results in more stable parameter estimates and typically increases the performance of the chosen measure of accuracy yet increases the chance that the model is tested on a nonrepresentative selection of cases. In contrast, allocating too much data for testing can leave the model poorly trained. A common allocation tradeoff used in practice is a proportion of 2:1 for training and testing. Another technique, used especially in situations in which data are limited, is cross-validation. Cross-validation divides data into multiple partitions (or folds), where each fold in turn is held out treated as the test set and the rest are used for training, and performance results are averaged across each test fold. Common used partitions are 5-fold, 10-fold, and leave-one-out cross-validation. Testing and cross-validation are not mutually exclusive, and both may be used to increase model robustness. These algorithms can be powerful tools for detecting previously unknown patterns in multidimensional data that may not be prima facie detectable by humans and also can be used to generate labels to subsequently train a supervised model. When different results are obtained from multiple approaches, we will take the overlap of results from different methods to reduce the probability of false positives. We will construct the predictive model using integrated advanced factors. Linear kernel in all algorithms will be used for our binary classification.

8. REFERENCES

1) Emblem, K. E., Pinho, M. C., Zollner, F. G., Due-Tonnessen, P., Hald, J. K., Schad, L. R., . . . Bjornerud, A. (2015). A generic support vector machine model for preoperative glioma survival associations. *Radiology, 275*(1), 228-234. doi:10.1148/radiol.14140770

2) Kaufman, G. I., DiFerdinando, G. T., Jr., & Gottesman, S. E. (1991). An evaluation of the use of the Statewide Planning and Research Cooperative System of New York State as a resource planning tool for HIV infection. *Am J Public Health, 81*(2), 215-217.
3) Khuri, S. F., Daley, J., Henderson, W., Hur, K., Demakis, J., Aust, J. B., . . . Stremple, J. F. (1998). The Department of Veterans Affairs' NSQIP: the first national, validated, outcome-based, risk-adjusted, and peer-controlled program for the measurement and enhancement of the quality of surgical care. National VA Surgical Quality Improvement Program. *Ann Surg, 228*(4), 491-507.
4) Machado, G. C., Ferreira, P. H., Yoo, R. I., Harris, I. A., Pinheiro, M. B., Koes, B. W., . . . Ferreira, M. L. (2016). Surgical options for lumbar spinal stenosis. *Cochrane Database Syst Rev, 11*, CD012421. doi:10.1002/14651858.CD012421
5) Matz, P. G., Meagher, R. J., Lamer, T., Tontz, W. L., Jr., Annaswamy, T. M., Cassidy, R. C., . . . Witt, J. P. (2016). Guideline summary review: An evidence-based clinical guideline for the diagnosis and treatment of degenerative lumbar spondylolisthesis. *Spine J, 16*(3), 439-448. doi:10.1016/j.spinee.2015.11.055
6) Memarian, N., Kim, S., Dewar, S., Engel, J., Jr., & Staba, R. J. (2015). Multimodal data and machine learning for surgery outcome prediction in complicated cases of mesial temporal lobe epilepsy. *Comput Biol Med, 64*, 67-78. doi:10.1016/j.compbiomed.2015.06.008
7) Moisi, M. D., Page, J., Gahramanov, S., & Oskouian, R. J. (2015). Bullet Fragment of the Lumbar Spine: The Decision Is More Important Than the Incision. *Global Spine J, 5*(6), 523-526. doi:10.1055/s-0035-1566231
8) Obermeyer, Z., & Emanuel, E. J. (2016). Predicting the Future - Big Data, Machine Learning, and Clinical Medicine. *N Engl J Med, 375*(13), 1216-1219. doi:10.1056/NEJMp1606181
9) Piper, K., Algattas, H., DeAndrea-Lazarus, I. A., Kimmell, K. T., Li, Y. M., Walter, K. A., . . . Vates, G. E. (2017). Risk factors associated with venous thromboembolism in patients undergoing spine surgery. *J Neurosurg Spine, 26*(1), 90-96. doi:10.3171/2016.6.SPINE1656
10) Senders, J. T., Staples, P. C., Karhade, A. V., Zaki, M. M., Gormley, W. B., Broekman, M. L. D., . . . Arnaout, O. (2018). Machine Learning and Neurosurgical Outcome Prediction: A Systematic Review. *World Neurosurg, 109*, 476-486 e471. doi:10.1016/j.wneu.2017.09.149
