# Supplementary material for: Sepsis and septic shock after craniotomy: Predicting a significant patient safety and quality outcome measure
Source: PLoS One. 2020 Sep 17;15(9):e0235273. doi: 10.1371/journal.pone.0235273 (PMC7498000; doi:10.1371/journal.pone.0235273)
Supplement: S3 File — (PDF) [file pone.0235273.s004.pdf]

UNIVERSITY of ROCHESTER

APPROVAL

November 27, 2018

The RSRB approved the following submission from 11/27/2018 to 11/26/2019 inclusive. This approval is effective as of 11/27/2018.

Type of Review:	Initial Study
Title:	Clinical outcome and Complication Prediction of Brain and Spine Surgery Using Machine Learning Model
Investigator:	Yan Li
Study ID:	STUDY00003122
Funding:	None
Regulatory Findings Consent:	Waiver or alteration of consent process
Regulatory Findings HIPAA:	Waiver or alteration of HIPAA
Documents Reviewed:	Protocol.pdf

This study was reviewed and approved under the OHSP and UR policies, and in accordance with Federal regulation 45 CFR 46 under the University's Federal-wide Assurance (FWA00009386). As the Principal Investigator, you are responsible for ensuring compliance with Policy 901 Investigator Responsibilities. Click here for the Summary of Responsibilities for Investigators Conducting Non-FDA Regulated Research. Also, any unanticipated problems involving risks to subjects or others (including unexpected deaths, hospitalizations or serious injuries, breach of confidentiality, loss of privacy) must be reported according to Policy 801 Reporting Research Events.

Association for the Accreditation of Human Research Protection Programs, Inc. Full AAHRPP Accreditation
